# Phosphorylated MAPK11 promotes the progression of clear cell renal cell carcinoma by maintaining RUNX2 protein abundance

**DOI:** 10.1111/jcmm.17870

**Published:** 2023-07-31

**Authors:** Xiandong Song, Changming Dong, Xiaojun Man

**Affiliations:** ^1^ Department of Urology The First Hospital of China Medical University Shenyang Liaoning China; ^2^ Department of Urology The First Hospital of China Medical University Shenyang Liaoning China

**Keywords:** clear cell renal cell carcinoma (ccRCC), MAPK11, P‐MAPK11, RUNX2, ubiquitination

## Abstract

Previous studies have demonstrated that mitogen‐activated protein kinase 11 (MAPK11) functions as an important point of integration in signalling transduction pathways and controlling endocellular processes, including viability of cells, differentiation, proliferation and apoptosis, through the sequence phosphorylation of the substrate protein Ser/Thr kinase protein cascade. Though MAPK 11 plays an important role in various tumours, especially in the invasive and metastatic processes, its expression and molecular mechanism in clear cell renal cell carcinoma (ccRCC) remain unclear. Runt‐associated transcription factor 2 (RUNX2), a main transcription factor for osteoblast differentiation and chondrocyte maturation, has high expression in a number of tumours. In this study, the mRNA and protein levels of targeted genes in ccRCC tissues and adjacent tissues are analysed using the Cancer Genome Atlas (TCGA) database and western blotting. The ccRCC cell proliferation was measured with colony formation and EdU assay, and cell migration was examined through transwell assay. The interactive behaviour between proteins was detected with immunoprecipitation. Half‐life period of RUNX2 protein was measured with cycloheximide chase assay. The results of the study indicated overexpression of MAPK11 and RUNX2 in ccRCC tissues and cell lines. MAPK11 and RUNX2 promoted the ccRCC cell proliferation and migration. Additionally, physical interaction took place between RUNX2 and P‐MAPK11, which functioned to sustain the stability of RUNX2 protein. The high expression of RUNX2 could neutralize the functional degradation in MAPK11. And the outcomes of the study suggest that the P‐MAPK11/RUNX2 axis may be used as a potential therapeutic target of ccRCC.

## 1 INTRODUCTION

Renal cell carcinoma (RCC) ranks the sixth commonest carcinoma in the male population and 10th in the female population globally, which account for 5% and 3% respectively among all malignancies clinically.^1^ While the diagnostic and managerial techniques for RCC have been improved in the past two decades, RCC remains one of the leading causes of death. An increasing number of cases of RCC are identified accidentally during routine imaging intended for other diseases.^2^ Clear cell renal carcinoma (ccRCC) is the commonest subcategory of RCC. Surgical treatment of ccRCC can remove the diseased tissue, but relapse and metastasis are found in about 40% of postoperative ccRCC patients, and poor prognostic effects are particularly found in patients with advanced and metastatic disease, and merely 12% could reach a survival time of 5 years after diagnosis.^3^, ^4^ Given that conventional chemoradiotherapy does not work for ccRCC,^5^ the development of new therapeutic strategies is imminent both academically and clinically.

Mitogen‐activated protein kinases (MAPKs) functions as an important point of integration in signalling transduction pathways and controlling endocellular processes, including viability of cells, differentiation, proliferation and apoptosis, through the sequence phosphorylation of the substrate protein Ser/Thr kinase protein cascade. The tri‐tiered activation structure is composed of MAPK Kinase Kinase (MAP3K), phosphorylation of a MAPK Kinase, dual phosphorylation of MAPK proteins.^6^ P38 MAPK contains four isoforms p38α, p38β, p38δ and p38γ. They can promote the cell growth and motility and are highly homologous.^7^, ^8^ The expressions of p38α and p38β, which share approximately 75% homology, are ubiquitous, while the expressions of p38δ and p38γ are differential in the tissues.^9^ Overexpression of MAPK11 has been detected in a number of malignancies. Furthermore, MAPK11 plays a crucial role in the occurrence, invasion and migration of cancerous cells in breast carcinoma, endometrial carcinoma, hepatocellular carcinoma and other types of malignancies.^10^, ^11^, ^12^ Nevertheless, the impact of p38β (MAPK11) on ccRCC remains unclear and requires further exploration.

RUNX2 is a key transcription factor in osteogenesis, performs an important function in osteoblast differentiation.^13^, ^14^ Studies have suggested that RUNX2 is associated with malignancy progression, functioning in the invasive and migrating behaviour of a number of carcinomas, including cancers in prostate, bladder and mantle cell lymphoma.^15^, ^16^, ^17^ The mechanism of RUNX 2 in ccRCC remains unclear and requires further exploration.

Studies indicate p38β is also related to a few transcription factors.^18^ MEFs (Myocite Enhancer Factors, for instance, play the regulatory role in various aspects of cells, such as apoptosis, proliferation, differentiation as well as migration and metabolism.

Phosphorylation of MEF2A and MEF2C by p38β occurs in the transcriptional activation domain of MEF2 and stimulates the transcriptional activity in vivo.^19^ The absence of MAPK14 is apt to result in decline of the protein level in RUNX2.^20^

The results of this study showed that P‐MAPK11 and RUNX2 were overly expressed in ccRCC, and P‐MAPK11 could have physical interaction with RUNX2. In addition, P‐MAPK11 helps keep RUNX2 protein stable by blocking its pathway of ubiquitination degradation.

## 2 MATERIALS AND METHODS

### 2.1 Bioinformatics analyses

The Cancer Genome Atlas (TCGA) was used for the analysis of the mRNA expression levels of particular genes in ccRCC tissues. Relevant information to TCGA is available on this website: http://gepia.cancer‐pku.cn.

### 2.2 Tissue samples

The tissue samples were derived from 32 patients hospitalized at the First Hospital of China Medical University from November 2021 to February 2022. All tissues were histologically examined and diagnosed as ccRCC. The study won approval from the hospital and all patients signed a consent form.

### 2.3 Cell lines and cell culture

All cells were incubated in strict accordance with the manufacturer's protocol. Different types of cells were cultivated in different culture mediums: RPMI‐1640 medium for human ccRCC cell lines, McCoy's 5A medium for CAKI‐1 cells, MEM medium for ACHN cells and DMEM/F12 medium for HK‐2 cells. Fetal bovine serum (10%, HyClone) was added to the mediums. All cell lines were obtained from Shanghai‐based Chinese Academy of Sciences and humidified incubated at 37°C in the required environment (95% air and 5% CO2). The cells at 80% confluence were treated with 1× PBS and trypsinized at 37°C for a requested duration to facilitate cell passage cultivation.

Losmapimod (GW856553X) and cycloheximide (CHX) were bought from MCE (MedChemExpress). The cells were cultivated with Losmapimod (GW856553X) (20 nmol/L) for at least 24 h and then the protein extraction was performed. Following the treatment of cells with CHX (100 μg/mL), cellular protein was collected for immunoblotting analysis to judge the half‐life period of RUNX2.

### 2.4 Western blotting

RIPA buffer combined with protease inhibitor cocktail were applied to lysed tissues and cells, and a BCA protein assay kit was utilized for protein concentration detection. SDS‐PAGE (140 V, 10%) was employed for the separation of protein fractions. A Mini Transblot Cell device was selected to transfer resolved protein (350 mA) to PVDF membranes (0.2 μm). The membranes were blocked in a capsule with fat‐free milk (5%) at 37°C for 60 minutes and cultured at 4°C overnight with antibodies. The antibodies included anti‐RUNX2 (1:1000, 12,556) and anti‐β‐tubulin (1:1000, 2128S) from Cell Signalling Technology, anti‐MAPK11 (1:1000, PHT5450M) and anti‐P‐MAPK11 (1:1000, T40076) from Abmart. The membranes were afterwards cultured with the anti‐rabbit secondary antibody for 60 min at 37°C. Quantification of the immunoblots was performed using ImageJ software (version 1.51).

### 2.5 Quantitative real‐time PCR

The separation of aggregate RNA was via TRIzol reagent and PrimeScriptTM RT reagent Kit from Takara Biotechnology Dalian and Takara in Japan. Reverse transcription process strictly followed the manufacturer's protocol. Quantitative PCR in real‐time was evaluated with the Swiss system of LightCycler™480 II Relative quantification was conducted using. The forward and reverse primers were as such: MAPK11 (5′‐ GGAGAACTGGTCGCCATCAAG ‐3′ and 5′‐ ACATTGGGTTCTCCTCGGACC ‐3′); RUNX2 (5’‐GCGCATTCCTCATCCCAGTA‐3′ and 5’‐GGCTCAGGTAGGAGGGGTAA‐3′); GAPDH (5’‐GGAGCGAGATCCCTCCAAAAT‐3′ and 5’‐GGCTGTTGTCATACTTCTCATGG‐3′).

### 2.6 Lentiviral transduction

Lentiviral‐based plasmids for MAPK11 knockdown and those for the overexpression of RUNX2 were synthesized by and purchased from Shanghai‐based GeneChem. MAPK11 knockdown stable cell lines were produced with the lentivirus vector containing short hairpin (sh) RNAs targeting MAPK11 (shRNA‐MAPK11) and negative control vector (shRNA‐Ctrl). Virosome that contains RUNX2 fragment in full length were deployed to produce LV‐RUNX2. The negative control vectors (LV‐Ctrl) were produced as well. Lentivirus infection technology was used to make stable cell lines and the infected cells were selected after treatment of the growth medium with 10 μg/mL puromycin (for no less than four passages).

### 2.7 Co‐immunoprecipitation assay

RIPA buffer combined with protease inhibitor cocktail were employed to lysed cells and centrifugation was performed with the cells (12,000 *g*) for 20 min at 4°C for immunoprecipitation. Subsequently, the cell lysate was cultured with a mixture of antibodies composed of anti‐P‐MAPK11, anti‐RUNX2 and anti‐IgG overnight at 4°C in rotation. The antibodies were obtained from Abmart or Cell Signalling Technology, respectively. The pyrolysis solution underwent rotation for 4 h with magnetic beads in. After the beads were taken out with a magnetic rack, protein loading buffer (5×) was added and denatured at 100°C for 10 min. Western blotting was carried out subsequently.

### 2.8 Colony formation assays

Trypsinization treatment was performed on cells growing to 80% confluence were trypsinized. The cells were removed to a newly prepared medium in single‐cell suspension. Dilution‐treated cells were seeded on six‐well plates, each plate containing 500 cells. They were then cultivated at 37°C with 5%CO2 for 10 days, and subsequently treated with crystal violet staining solution for 10 min. ImageJ software were drawn upon for the quantification of Colony areas.

### 2.9 Transwell assays

Serum starvation was performed for 24 h and the cells were then seeded in transwell inserts (2 × 10^4^ cells, 8‐μm pore size, Corning). Then, cells treated with serum‐free medium (200‐μL) were cultured on the upper layer of the 24‐well plates, and the solution was (10% FBS, 600 μL) was infused into the lower layer. After 12‐h incubation in dark environment, crystal violet staining was performed for 10 min to the chamber. Cells in the upper layer were collected with buccal swabbing technique and observed for migrating effects using ImageJ.

### 2.10 EdU Assay

Cells were placed in six‐well plates with EdU (BeyoClick™, EDU‐488) for labelling. Culture medium was taken away followed by EdU staining after being labelled, the cells were treated for half an hour with click reaction cocktail in dark environment at normal room temperature in conformity with the manufacturer's guidelines. A fluorescent microscope (Olympus Corporation) was used for imaging.

### 2.11 Statistical analysis

Data analysis was performed with SPSS 21.0 software and GraphPad Prism version 9.0 (La Jolla, CA, USA). All the statistics were expressed as mean ± SD. The difference with *p* < 0.05 is statistically significant.

## 3 RESULTS

### 3.1 Phosphorylated MAPK11 and RUNX2 are Highly Expressed in ccRCC tissues and ccRCC cell lines

**FIGURE 1:**
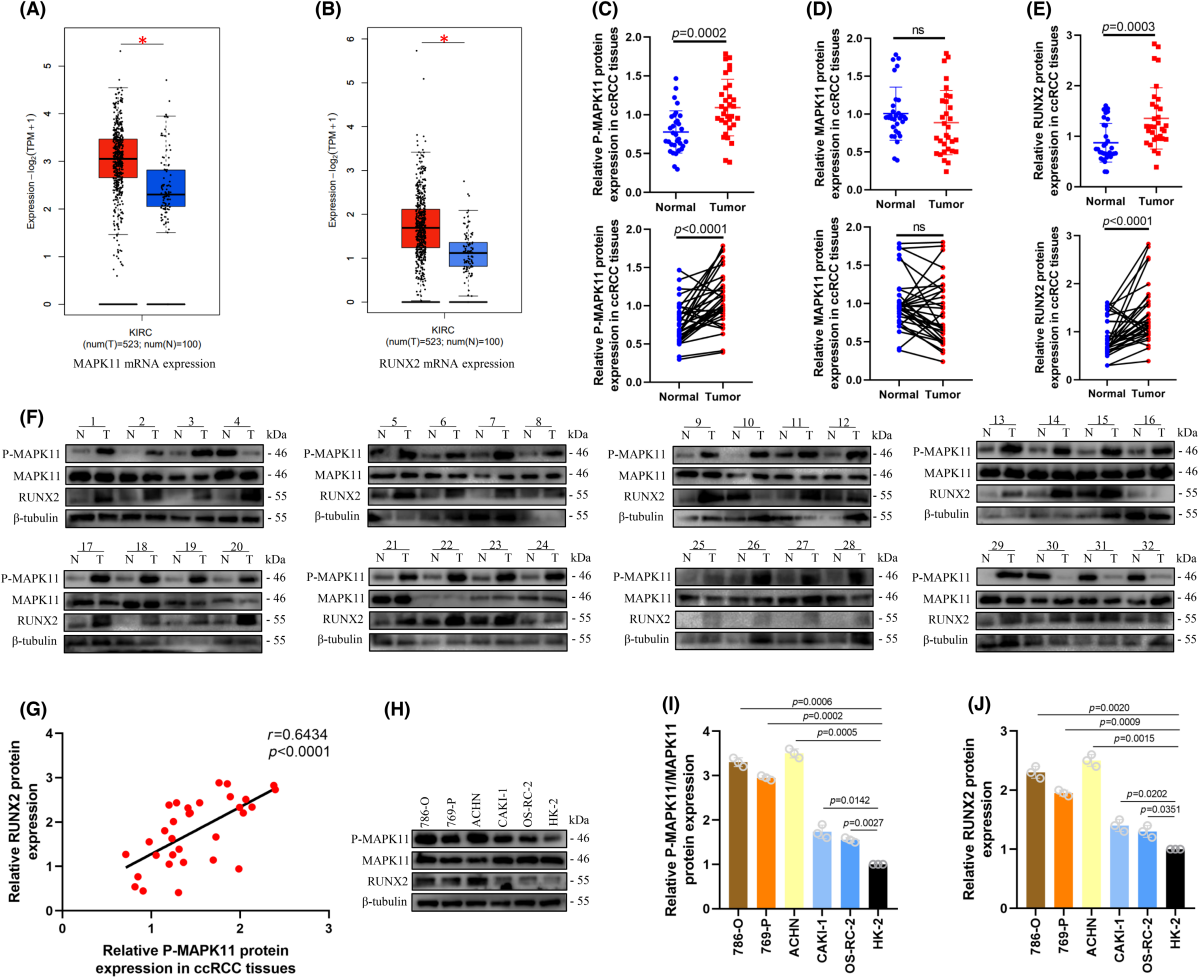
Phosphorylated MAPK11 and RUNX2 are highly expressed in ccRCC tissues and cell lines. (A, B) The transcriptional expression level in TCGA datebase. (C–F) Higher expression in cancerous tissues than in normal tissues. (G) Positive correlation between RUNX2 and P‐MAPK11 protein expression in ccRCC tissues. (H–J) Protein expression among ccRCC cell lines. The difference with *p* < 0.05 is statistically significant.

The transcription levels of MAPK11 and RUNX2 in ccRCC from TCGA database were analysed drawing upon the Gene Expression Profiling Interactive Analysis. And it was revealed that the transcription level of MAPK11 and RUNX2 was upregulated in ccRCC (Figure 1A,B). P‐MAPK11 is the primary activation form of MAPK11, and the protein expression level of RUNX2, MAPK11 and P‐MAPK11 was analysed in 32 pairs of ccRCC tissues and neighbouring normal tissues to investigate the clinical role of ccRCC. Western blotting analysis suggested that the protein expression level in RUNX2 and P‐MAPK11 in cancerous tissues outstripped that in the healthy tissues. The protein expression in MAPK11 showed no considerable difference (Figure 1C–F). The expression of P‐MAPK11 and RUNX2 protein was closely related to Fuhrman grade but not to such factors as age, gender, the stage and size of tumour (Table 1). Because of the unavailability of advanced tumours, distant and lymphatic metastases were not observed in this study. Next, the correlation between P‐MAPK11 and RUNX2 protein expression level in 32 ccRCC samples was analysed and RUNX2 expression had positive correlation with P‐MAPK11 protein level (Figure 1G). In addition, compared with HK2 cell lines, high expression levels in P‐MAPK11 and RUNX2 were also observed in 786‐O, ACHN, 769‐P, OSRC‐2 and CAKI‐1 cell lines (Figure 1H–J). These findings suggest that P‐MAPK11 and RUNX2 were overly expressed in cell lines and diseased ccRCC tissues.

**TABLE 1:** Correlation between P‐MAPK11 and RUNX2 expression and the clinicopathological parameters of 32 ccRCC patients.

			P‐MAPK11			RUNX2		
Variables	Group	N	Low	High	*p‐*value^a^	Low	High	*p‐*value^a^
Gender	Male	14	4	10	>0.99	3	11	>0.99
	Female	18	5	13		5	13	
Age	≥60	15	5	10	0.6989	4	11	>0.99
	<60	17	4	13		4	13	
T stage	T1/T2	12	3	9	>0.99	2	10	0.6757
	T3/T4	20	6	14		6	14	
Tumour size (cm)	≥5	10	2	8	0.6808	2	8	>0.99
	<5	22	7	15		6	16	
Fuhrman grade	1/2	26	4	22	0.0033*	3	23	0.0015*
	3/4	6	5	1		5	1	
Distant metastasis	Negative	30	8	22	0.4899	7	23	0.4435
	Positive	2	1	1		1	1	
Lymphatic invasion	Positive	0			—			—

*Note*: *p‐*value^a^ was determined by Pearson Chi‐square tests and *p < 0.05 was considered significant.

### 3.2 Downregulation of MAPK11/P‐MAPK11 Protein Inhibits ccRCC Proliferation and Migration

**FIGURE 2:**
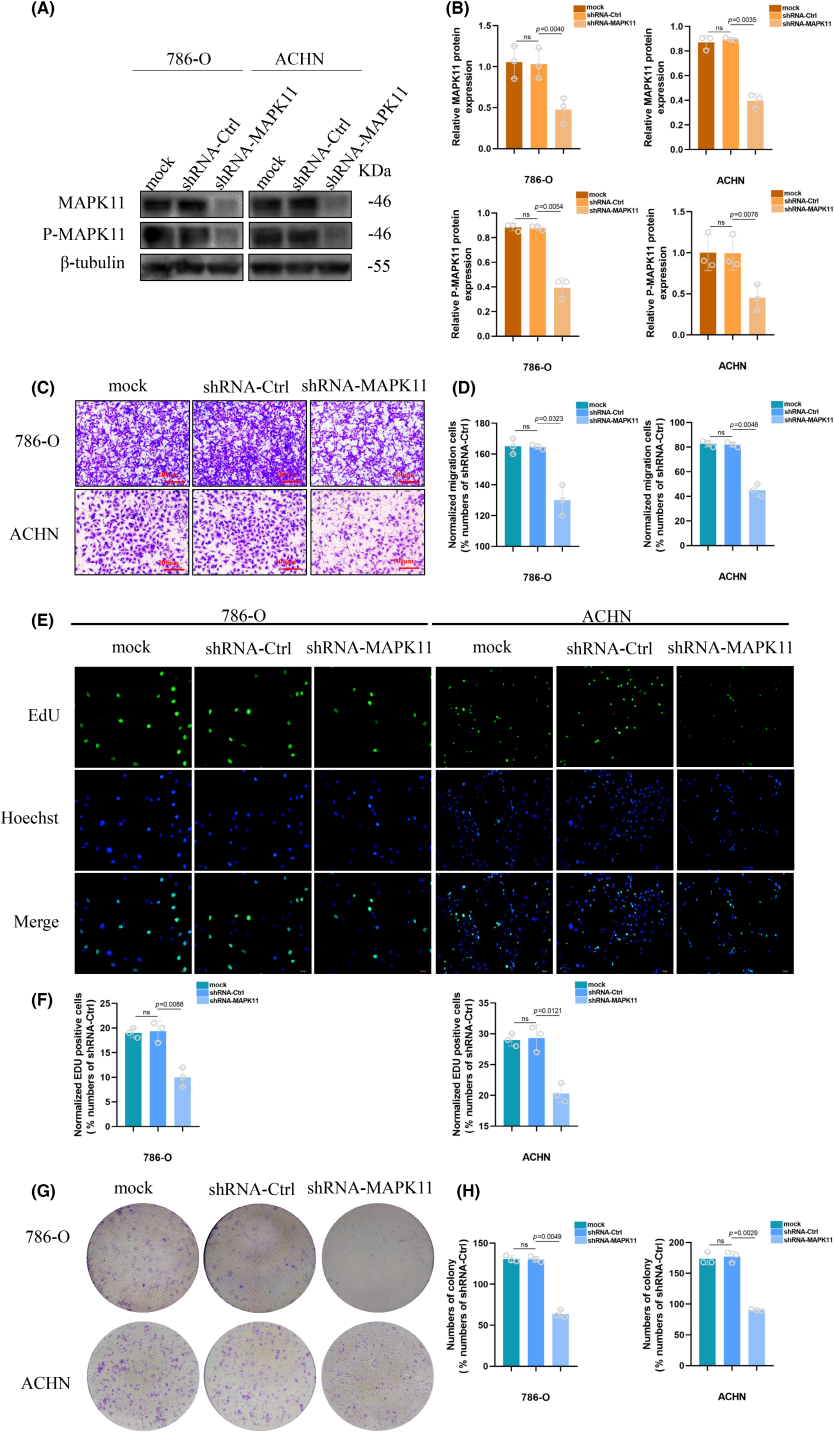
Downregulation of MAPK11/P‐MAPK11 Protein Inhibits ccRCC Proliferation and migration (in 786‐O and ACHN cell lines). (A, B) Protein level under western blotting in cells transfected with shRNA‐MAPK11. (C, D) Migrating capability under transwell assay (magnification×20). (E, F) Proliferating capability under EdU assay (magnification×200). (G, H) Colony formation capability under colony formation assay. The difference with *p* < 0.05 is statistically significant.

MAPK11 is a gene that plays a vital part in tumour cell proliferation and migration.^21^, ^22^, ^23^ MAPK11 knockdown stable cell lines were generated to functionally dissect the potential role of MAPK11 in the proliferation and migration of ccRCC cells. As showed by Western blotting (Figure 2A,B), the protein expression levels of MAPK11 and P‐MAPK11 were considerably diminished by shRNA‐MAPK11 in 786‐O and ACHN cells. Migration assay showed the cells' migration ability was impeded by depletion of MAPK11 (Figure 2C,D). EdU assay suggested that MAPK11 knockdown hindered cellular proliferation (Figure 2E,F). Similarly, colony formation assay showed the cells' colony formation ability was notably influenced in the absence of MAPK11 (Figure 2G,H). All in all, knockdown of MAPK11 could produce the effect of weakened activity and proliferating capability of ccRCC cells in vitro.

### 3.3 P‐MAPK11 Regulates the RUNX2 Protein Expression

**FIGURE 3:**
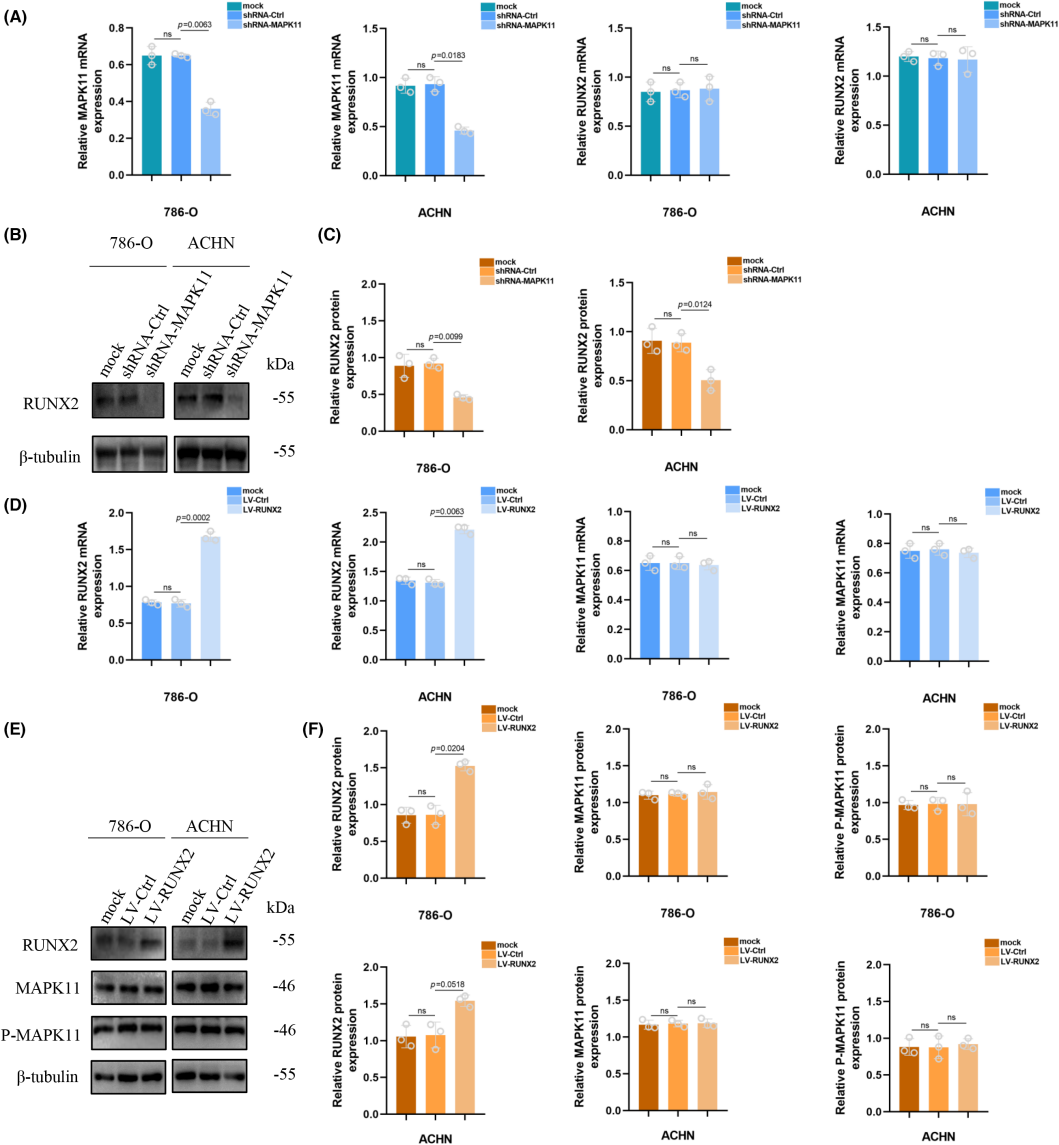
P‐MAPK11 Regulates the RUNX2 protein expression. (A) The mRNA level in 786‐O and ACHN cells transfected with shRNA‐MAPK11. (B, C) RUNX2 protein level in 786‐O and ACHN cells transfected with shRNA‐MAPK11. (D) The mRNA level in 786‐O and ACHN cells transfected with LV‐RUNX2. (E, F) Protein levels in 786‐O and ACHN cells transfected with LV‐RUNX2. The difference with *p* < 0.05 is statistically significant.

Previous studies have been shown that MAPK14 depletion could result in a decline in RUNX2 protein level.^20^, ^24^ Therefore, stable MAPK11 knockdown ccRCC cell lines were prepared for the purpose of determining whether P‐MAPK11 could regulate the RUNX2 protein in ccRCC cells in our study. When MAPK11 was absent, the mRNA expression level of MAPK11 dropped considerably, while in the expression of RUNX2 mRNA, no change in statistical significance occurred (Figure 3A). It was indicated that RUNX2 protein expression dropped significantly (Figure 3B,C). The ccRCC cell lines overexpressing RUNX2 were used for determining the correlation between RUNX2 and the expression of MAPK11 and P‐MAPK11. No significant change showed in the mRNA expression of MAPK11 as RUNX2 was overly expressed (Figure 3D). And western blotting revealed that increased expression of RUNX2 did not cause change in the protein levels of MAPK11 and P‐MAPK11 (Figure 3E, F). These results showed that MAPK11 or P‐MAPK11 could regulate the RUNX2 protein expression, while the regulation mechanism remained to be explored.

### 3.4 P‐MAPK11 Regulated the RUNX2 Protein Expression and Maintained the Stability of the RUNX2 Protein

**FIGURE 4:**
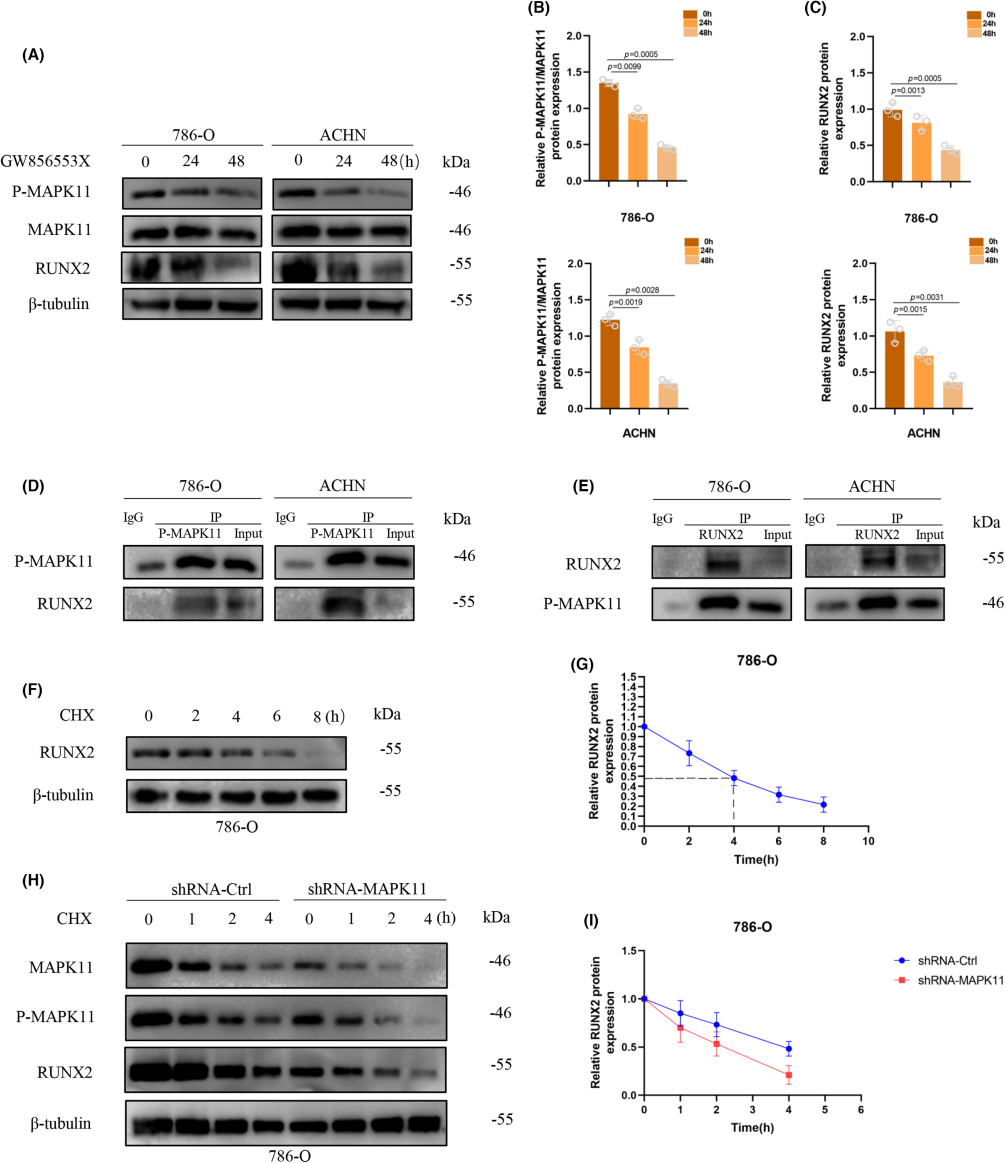
P‐MAPK11 Regulated the RUNX2 protein expression and maintained the stability of the RUNX2 protein. (A–C) Protein levels under western blotting after treatment with GW856553X. (D, E) Physical interaction under co‐immunoprecipitation assay. (F, G) Aggregate cellular lysates under western blotting analysis after treatment with cycloheximide (100 μg/mL). (H, I) Aggregate cellular lysates under western blotting analysis after treatment with cycloheximide (100 μg/mL). The difference with *p* < 0.05 is statistically significant.

To determine whether the decline of RUNX2 protein was a consequence of that of P‐MAPK11, the study selected the inhibitor Losmapimod (GW856553X) of P‐MAPK11, which could downgrade the phosphorylation level of MAPK11 while maintaining the protein level in MAPK11.^25^ Cells were harvested with Losmapimod (GW856553X) (20 nmol/L) for 0, 24, 48 h. As is shown in the western blotting (Figure 4A–C), RUNX2 protein dwindled with the decline of P‐MAPK11 protein level. To explore the regulatory role of P‐MAPK11 in RUNX2, we carried out co‐immunoprecipitation assay on two ccRCC cell lines 786‐O and ACHN. The results were shown in (Figure 4D,E). Studies demonstrated that RUNX2 protein is apt to be degradated in ubiquitin–proteasome‐dependent pathway.^26^, ^27^ Cycloheximide chase assay was employed for the analysis of half‐life of the RUNX2 protein. 786‐O cell underwent CHX treatment (100 μg/mL) for 0, 2, 4, 6 and 8 h. It was found that protein content of RUNX2 was degradated to approximately 50% at 4 h in comparison with that at 0 h (Figure 4F,G). Hence, the conclusion was that the half‐life period of the RUNX2 protein was 4 h. CHX treatment (100 μg/mL) was also performed to cells with stable expression of shRNA‐Ctrl and shRNA‐MAPK11 for a specific duration and then the protein of cells was collected for immunoblotting analysis. As MAPK11/P‐MAPK11 decreased markedly, the half‐life period of RUNX2 protein decreased significantly (Figure 4H,I). The findings indicate that MAPK11/P‐MAPK11 could stabilize RUNX protein presumably by blocking its pathway of ubiquitination degradation.

### 3.5 RUNX2 restored the effect of MAPK11 and P‐MAPK11 in part in ccRCC progression

**FIGURE 5:**
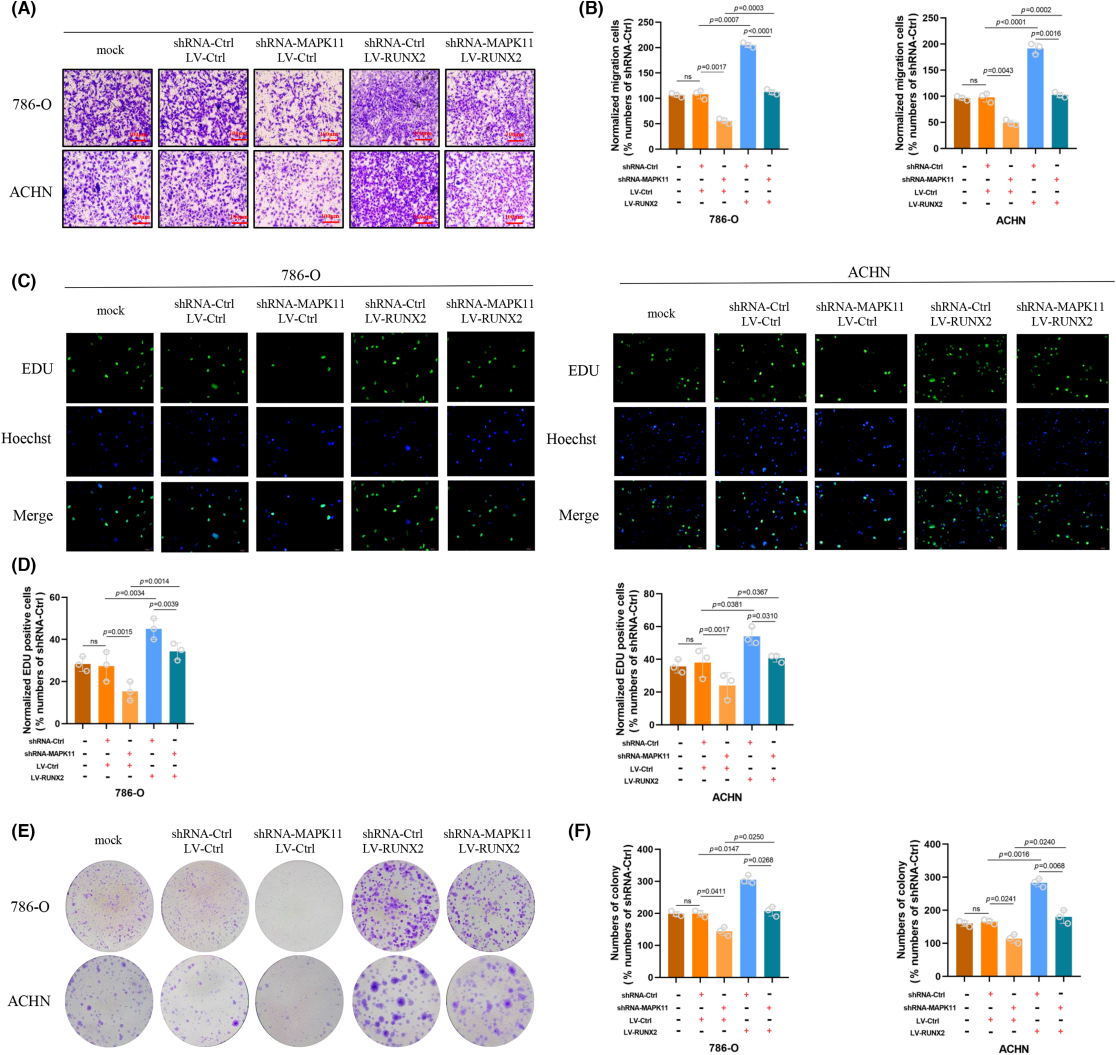
RUNX2 partially restored the effect of MAPK11 and P‐MAPK11 in the progression of ccRCC (in 786‐O and ACHN cell lines). (A, B) Migrating capability under transwell assay (magnification × 20). (C, D) Proliferating capability under EdU assay (magnification × 200); (E, F) Colony formation ability under colony formation assay. The difference with *p* < 0.05 is statistically significant.

In order to explore the role RUNX2 played in MAPK11/P‐MAPK11‐mediated cellular proliferating, migrating and invasive behaviour of ccRCC, the MAPK11 knockdown cells were transfected with virosome that contains RUNX2 in full length (shRNA‐MAPK11/LV‐RUNX2). As shown by migration assay (Figure 5A,B), the overexpression of RUNX2 led to incomplete reverse in the diminishment of cellular migrating capability resulting from the decrease of MAPK11 and P‐MAPK11. Through EdU assay and colony formation assay, it was found that the deficiency of MAPK11 and P‐MAPK11 led to the impairment of the proliferating ability of ccRCC cells, which was restored partially when RUNX2 was overly expressed (Figure 5C–F). The above data showed that ccRCC progression could be promoted to some extent through the stabilization of RUNX2 protein.

## 4 DISCUSSION

This study found no increase in the level of transcription and translation of MAPK11 in ccRCC. Protein expression in P‐MAPK11 and RUNX2 were markedly elevated in ccRCC cell lines and tissues. Apart from that, the overexpression of P‐MAPK11 and RUNX2 in protein levels correlated with the Fuhrman grade of ccRCC tissues. The expression of P‐MAPK11 was closely linked to protein expression of RUNX2 in diseased tissues. This study indicated that MAPK11 and RUNX2 could be a biomarker and targeted therapy for ccRCC in clinical practice. P‐MAPK11 could have physical interaction with RUNX2, and hence promote cell proliferation and migration in the cancer in question. P‐MAPK11 demonstrated a regulatory function in the protein expression of RUNX2 and a stabilizing role in RUNX2 protein via ubiquitination degradation pathway.

It has been reported that a variety of proteins and substrates could be phosphorylated by P38 MAPK including many transcription factors.^28^ And MAPK11 is thought that it relates to the proliferating, invasive and metastatic behaviour of a variety of cancerous cells.^29^, ^30^, ^31^ Aside from that, this study examined the mechanism of P‐MAPK11 in ccRCC cells in 786‐O and ACHN. When shRNA‐MAPK11 diminished the abundance of P‐MAPK11 protein, the cellular proliferating, migrating and clone‐forming capabilities showed significant decline. In view of the fact that P‐MAPK11 is the main functional form of MAPK11, the overexpression of the protein could be found in both ccRCC tissues and cell lines. P‐MAPK11 was presumably conducive to the development of ccRCC.

RUNX2 could push osteoblast phenotype to change from immaturity to maturity and hence promote skeletal development^32^, ^33^ Additionally, RUNX2 plays the crucial role in the migrating, invasive and metastatic process of a variety of cancers.^34^, ^35^, ^36^, ^37^ Recently, the correlation between MAPKs and RUNX2 has been recorded in different tumours.^38^, ^39^ And study has shown that RUNX2 could be phosphorylated and activated by P38 MAPK.^40^ Our study revealed that the expression of RUNX2 became lower because of the downregulation of MAPK 11, while mRNA level remained largely unchanged in RUNX2 expression. This finding indicated that MAPK11 exerted an impact on RUNX2 transcription and protein expression. Subsequently, the phosphorylation inhibitor of MAPK11 was employed for the exploration of the mechanism of the downregulation of RUNX2 protein. It was revealed that the protein expression level of P‐MAPK11 was decreased and RUNX2 protein level was also diminished. It could be concluded that P‐MAPK11 served the regulatory role for stabilizing RUNX2 protein. Meanwhile, co‐immunoprecipitation assay also indicated that RUNX2 protein, which was ubiquitinylated prior to proteasomal degradation,^41^, ^42^ could have physical interaction with P‐MAPK11 protein. Without the presence of MAPK11 protein, RUNX2 protein degraded more quickly. All these findings pointed to the probability that RUNX2 activated SCD1 protein and sustained its stability. In addition, the protein rescue experiment demonstrated that the growth and migrating behaviour of ccRCC cells improved.

In conclusion, P‐MAPK11 could intermodulate with RUNX2 to facilitate the progress of ccRCC. Our research outcomes reveal that RUNX2 is a substrate of P‐MAPK11, and P‐MAPK11 could play a preventative role through binding. As a consequence, it is in need of exploring the regulatory mechanism of P‐MAPK11 in the ubiquitination pathway degradation of RUNX2.

## AUTHOR CONTRIBUTIONS

**Xiandong Song:** Conceptualization (lead); data curation (lead); formal analysis (lead); funding acquisition (lead); investigation (lead); methodology (lead); project administration (lead); resources (equal); software (equal); writing – original draft (lead); writing – review and editing (lead). **Changming Dong:** Supervision (equal). **Xiaojun Man:** Validation (equal); visualization (equal).

## FUNDING INFORMATION

No funding from any individual or organization is involved in this study.

## CONFLICT OF INTEREST STATEMENT

We declare that our work involves no conflict of interest of any nature with any individual or professional organization.

## Data Availability

The data sets involved in this study are obtained from relevant authors at the reasonable request.
